# Utilizing decision tree machine learning model to map dental students’ preferred learning styles with suitable instructional strategies

**DOI:** 10.1186/s12909-023-05022-5

**Published:** 2024-01-11

**Authors:** Lily Azura Shoaib, Syarida Hasnur Safii, Norisma Idris, Ruhaya Hussin, Muhamad Amin Hakim Sazali

**Affiliations:** 1 Department of Paediatric Dentistry & Orthodontics, Faculty of Dentistry, Universiti Malaya, 50603 Kuala Lumpur, Malaysia; 2 Department of Restorative Dentistry, Faculty of Dentistry, Universiti Malaya, 50603 Kuala Lumpur, Malaysia; 3 Department of Artificial Intelligence, Faculty of Computer Science and Information Technology, Universiti Malaya, 50603 Kuala Lumpur, Malaysia; 4 Department of Psychology, International Islamic University Malaysia, Jalan Gombak, 53100 Kuala Lumpur, Malaysia; 5 Department of Artificial Intelligence, Faculty of Computer Science and Information Technology, Universiti Malaya, 50603 Kuala Lumpur, Malaysia

**Keywords:** Student-centered learning, Learning styles, Dental undergraduates, Decision tree model instructional strategies

## Abstract

**Background:**

Growing demand for student-centered learning (SCL) has been observed in higher education settings including dentistry. However, application of SCL in dental education is limited. Hence, this study aimed to facilitate SCL application in dentistry utilising a decision tree machine learning (ML) technique to map dental students’ preferred learning styles (LS) with suitable instructional strategies (IS) as a promising approach to develop an IS recommender tool for dental students.

**Methods:**

A total of 255 dental students in Universiti Malaya completed the modified Index of Learning Styles (m-ILS) questionnaire containing 44 items which classified them into their respective LS. The collected data, referred to as dataset, was used in a decision tree supervised learning to automate the mapping of students' learning styles with the most suitable IS. The accuracy of the ML-empowered IS recommender tool was then evaluated.

**Results:**

The application of a decision tree model in the automation process of the mapping between LS (input) and IS (target output) was able to instantly generate the list of suitable instructional strategies for each dental student. The IS recommender tool demonstrated perfect precision and recall for overall model accuracy, suggesting a good sensitivity and specificity in mapping LS with IS.

**Conclusion:**

The decision tree ML empowered IS recommender tool was proven to be accurate at matching dental students’ learning styles with the relevant instructional strategies. This tool provides a workable path to planning student-centered lessons or modules that potentially will enhance the learning experience of the students.

## Background

Teaching and learning are fundamental activities in an educational institution. In the effort to develop a high-quality professional educational framework, it is important to look at students’ learning needs. Interaction between students and their learning environment can be determined through their LS. Studies suggest that mismatched students’ LS and IS planned by educators might have a negative impact on students’ learning such as reduced concentration level and becoming less motivated. This will indirectly affect the academic achievement of the students [1, 2].

IS are methods that educators apply to deliver knowledge and skills to students including assisting students in learning [3]. In general, a good educator plans a teaching strategy or IS that is most suited with the level of knowledge of the students, the concept being studied, and their stage of learning journey. Theoretically when LS and IS are matched, students will be able to organise and use specific range of skills to learn effectively. In general, a lesson plan will include several transitions across stages, such as from teaching input to guided practice or from guided practice to independent practice stages. In view of this, efficient educator will usually outline learning with the objective of shaping students’ knowledge and skills [4].

### Student-centred learning strategy

Growing demand for SCL has been observed in higher education settings including dentistry. The SCL strategy is designed to meet students’ learning needs. This can be accomplished, for example if students are actively engaged in learning activities whereby educators will act as facilitators and be accountable for valuable feedback. Providing learning materials and activities that fit students’ LS or preferences is said to be hypothetically able to improve students’ learning environment and promote positive learning experience [5].

### Understanding learning in dentistry

In general, the learning process of dental undergraduates is influenced by their clinical environment from various clinical procedures that they must perform and building effective communication skills with people. The aim of teaching is for the students to integrate basic dental science knowledge with dental clinical skills and to apply their knowledge in new clinical situations [6, 7]. An earlier study on the relationship between LS and IS showed that by adapting learning strategies mapping to preferred LS will assist in improving the education process [8]. The authors also encourage adapting various teaching and assessment methods to suit learners’ LS and needs.

### Learning styles models

It is an advantage for educators to apply the LS knowledge in helping them to design, develop, and implement teaching which will enhance learners in acquiring more knowledge and understanding of the subject. Several LS evaluation tools have been developed by researchers such as Kolb’s model of experiential learning, Felder-Silverman Learning Style Model (FSLSM) and Fleming’s VAK/VARK model [5, 9, 10]. These learning models are commonly used and the most researched learning models according to the literature. The present research work adopted the FSLSM to evaluate the LS of dental undergraduate students.

#### The Felder and Silverman learning style model

FSLSM is a widely used model which has assessed adaptive learning in the field of engineering. There are many published works in the field of health sciences including medicine, nursing, pharmacy, and dentistry that can be found using the FSLSM model [5, 11–13]. The instrument used to measure the LS dimensions in FSLSM is known as Index of Learning Styles (ILS) [8] which contains 44 items, assessing across the four LS dimension: processing(active/reflective), perception (sensing/intuitive), input (visual/verbal) and understanding (sequential/global) [14].

**Fig. 1:**
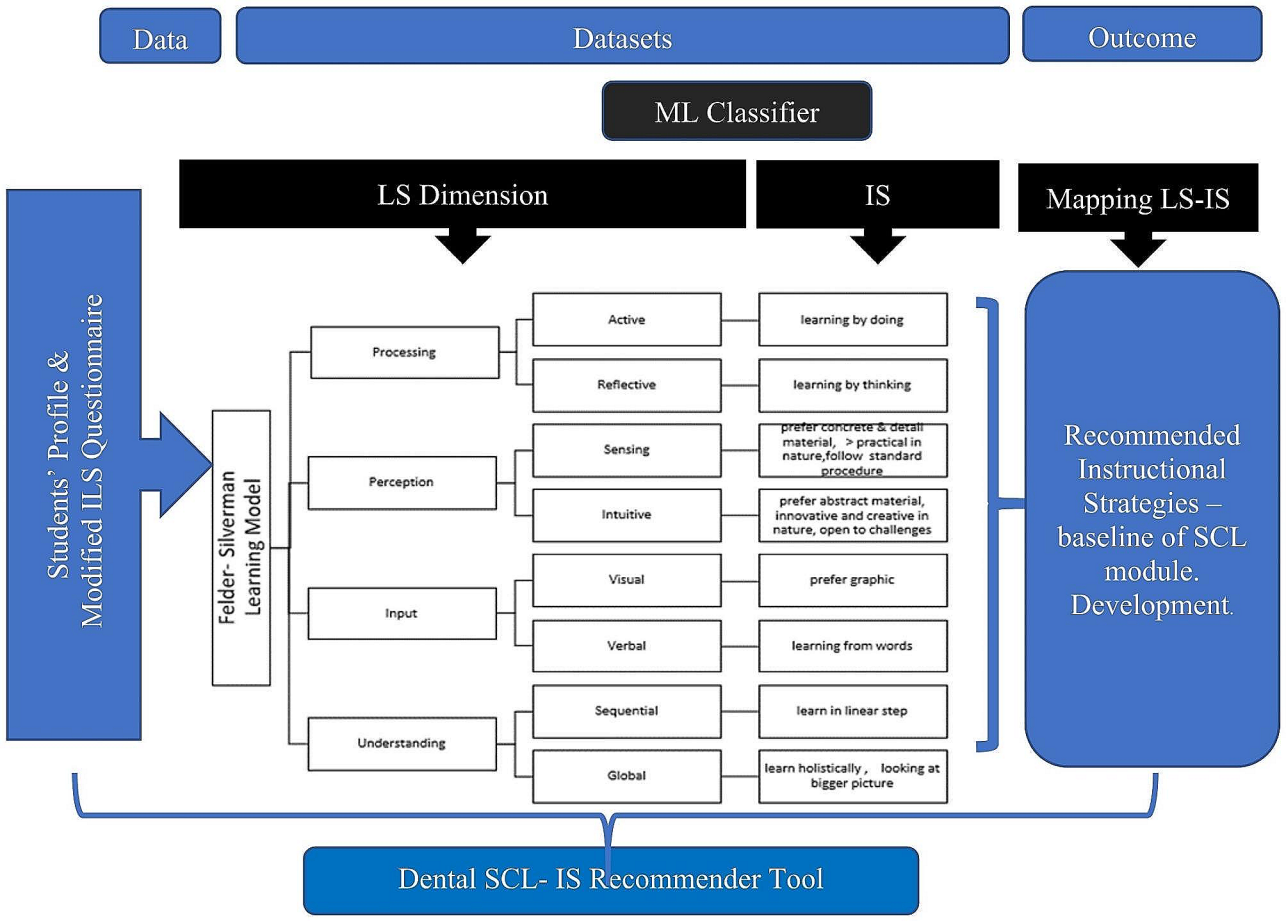
Development framework of student-centred learning IS recommender tool

As depicted in Fig. 1, each dimension of FSLSM will have one dominant preference. For example, in the “processing” dimension, students with “active” LS prefer to process information by interacting directly with the learning material, learning by doing and they tend to study in a group. “Reflective” LS refers to learning by thinking and prefer to work individually. The “perception” LS dimension can be classified with either “sensing” or /and “intuitive.” “Sensing” learners prefer more concrete information and practical procedures, facts orientation compared to their “Intuitive” counterpart who are more comfortable with abstract material, are more innovative and creative in nature. “Input” LS dimension consists of ‘visual” and “verbal” learners. Those with “visual “LS prefer learning through visual presentation such as diagrams, videos, or live demonstration whilst those with “verbal” LS prefer learning from words either in written or oral explanations. For “understanding” LS dimension, this type of learners can be classified as “sequential” and “global.” “Sequential learners prefer a linear thinking process, learn in incremental steps, meanwhile “global” learners are prone to have a holistic thinking process and always have a bigger picture of what they have learned.

### Utilizing machine learning in the development of student centered-learning module

#### Mapping students’ learning styles and instructional strategies

Of late, many researchers have started to work on data-driven approaches for automated detection involving development of new algorithms and models capable of interpreting a multitude of data [15, 16]. Depending on the data provided, supervised ML (Machine Learning) is able to generate patterns and hypotheses predicting future outcomes based on the construction of an algorithm [17]. In brief, the supervised ML technique manipulates the input data and trains the algorithm. Subsequently, it generates a perimeter to classify or predict the outcomes based on similar circumstances of the provided input data. The main advantage of supervised ML algorithm is its capability in setting ideal and desired outcomes [17].

Through data-driven approaches and the application of decision trees supervised model, automated detection of LS was made possible. Decision tree is reported to be widely applied in learning programs in various domains including health sciences [18, 19]. In this study, the model was trained specifically by the system developer to determine the students’ LS and recommend the best IS for it.

#### Instructional strategies recommender tool based on learning styles

This study was designed to strategize the IS delivery according to students’ LS towards application of SCL approach through the development of IS recommender tool mapping to LS. The development framework of the IS recommender tool as a strategy for SCL approach is illustrated in Fig. 1. The IS recommender tool is divided into two parts which include LS classification mechanism utilizing ILS and mapping of best fit IS for the students.

Specifically, the characteristics of the IS recommender tool include application of web technology and application of decision trees machine learning. The system developer enhanced user convenience and portability by adapting it to mobile devices such as mobile phones and tablets.

## Methodology

### Data collection

The experiments were carried out in two phases with voluntary participation of the undergraduate students in Faculty of Dentistry, Universiti Malaya. The participants answered the online m-ILS for dental students in English language. In the initial phase, the dataset of 50 students was used to train the decision trees machine learning algorithm. In the second phase of the development process, the dataset of 255 students was used to enhance the precision of the developed tool.

### Data collections

All participants were given an online briefing at the beginning of every phase according to the academic year in Microsoft Teams. Purpose of the study was explained and informed consent was obtained. All participants were given a link to access the m-ILS. Each student was assigned to answer all 44 items of the questionnaire. They were given one week to complete the modified ILS at their own convenience and space, during the semester break before the start of the academic semester. The m-ILS was modified based on the original ILS instrument to suit dental students. Resembling original ILS, it contains 44 items (a, b), evenly distributed with 11 items each to assess aspect of each dimension of the FSLSM.

### Steps of instructional strategies recommender tool development

In the initial stage of the tool development, the mapping was annotated manually by the researcher using the dataset of 50 dental students. The system was presented with a summation of the answer ‘a’ and ‘b’ according to FSLM. For each dimension, if a student chose ‘a’ for an answer, the LS was classified as either active /sensing/visual/ sequential and if ‘b’ was chosen for an answer, the student was assigned as reflective/ intuitive/verbal /global learner.

Following a workflow calibration between dental education researcher and the system developer, questions were selected according to the FSLSM domain and fed into ML models to predict each student’s LS. “Garbage in, garbage out” is a popular quote in the ML field where it emphasizes data quality. The input quality determined the accuracy and precision of the machine learning model. Through a feature engineering phase, a new feature selection was created which is a summation of the answers “a” and “b” according to the FSLSM. The item numbers for identification of LS are shown in Table 1.

**Table 1:** Learning style and item number mapping

Learning Style Dimension	Learning Style Classification (based on answer a / b summation)	Item number
Processing	Active/Reflective	1,5,9,13,17,32,25,29,33,37,41
Perception	Sensing/Intuitive	2,6,10,14,18,22,26,30,34,38,42
Input	Visual/Verbal	3,7,11,15,19,23,27,31,35,39,43
Understanding	Sequential/Global	4,8,12,16,20,24,28,32,36,40,44

From the answers, scores were calculated, and students’ LS were determined. For each student, the scores would range from 1 to 11. The scores 1 to 3 indicates a balance of learning preference within the same dimension, whilst 5 to 7 indicates a moderate preference showing that the students’ tendency to favor one learning environment compared to another within the same dimensions, and scores between 9 and 11 reflects a strong preference for one end or the other [8].

For each of the dimension, the LS were grouped according to “active,” “reflective,” and “balanced.” For example, a student belongs to “active” LS domain when he/she answers more ‘a’ than ‘b’ within the assigned items and the score exceeded a threshold of 5 for the specific items representing ‘processing’ LS dimension. However, the student would be classified as having “reflective” LS when he/she selected ‘b’ more than ‘a’ for the specific 11 questions (Table 1) with the score exceeding 5. Finally, the student is in a “balanced processing” LS if the score did not exceed score 5. The repetition of the classification processes was repeated for the other LS dimensions which were perception (active/reflective), Input (visual/verbal), and understanding (sequential/global).

### Application of decision tree model

Decision tree model has the ability to use different feature subset and decision rules at various stages of the classification process. It is considered as a popular tool for classification and prediction. It could be represented using a tree structure like flowchart [20] which has internal node that denotes a test on an attribute, each branch represents an outcome of the test, and each leaf node (terminal node) holds a class label.

A simple rule-based program was created to automate the calculation of the score and annotate each student’s LS based on their responses. The rule-based took the form of IF statements where ‘IF’ outlines the trigger, ‘THEN’ specifies the action to complete e.g.: ‘IF X happens THEN do Y’ (Liu et al. 2014). If the dataset showed to be relevant and the decision tree model was properly trained and evaluated, the approach can be an effective way to automate the process of LS and IS mapping.

**Fig. 2:**
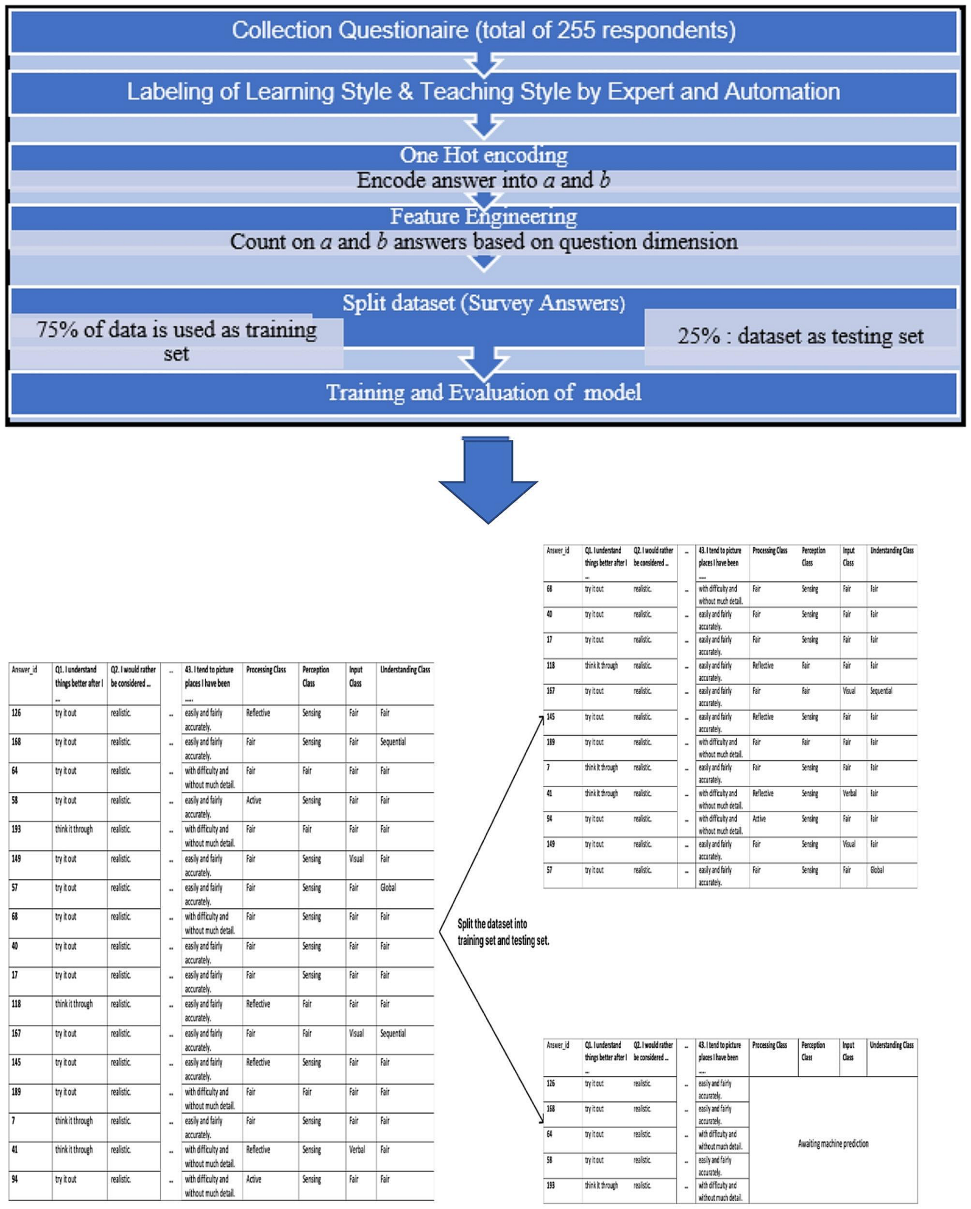
Student centered-learning IS recommender tool development process

During the second phase of development, the dataset was increased to 255 to increase accuracy of the recommender tool. The data set was split into a ratio of 1:4. For the test set, 25% (64) of the dataset was used and the remaining 75% (191) was used as the training set (Fig. 2). Split of dataset had to be done to prevent the model from learning and testing on the same dataset, which might cause the model to remember and not learn. The model learned on the training set and assessed the effectiveness of the test set, which the model had never seen the data before.

Following completion of the IS tool development, the application could classify the LS of a dental student based on their answers through a web-based interface. The web-based system of the IS recommender tool was built using Python programming language with Django framework as the backend. The libraries used in the development of this system are presented in Table 2.

**Table 2:** Packages extensively used in this study

Package Name	Description	Reference link
Numpy	NumPy support for multi-dimensional arrays and matrices, high-level mathematical functions to operate on these arrays.	https://numpy.org/
Pandas	Data manipulation and analysis	https://pandas.pydata.org/
Django	Django is a high-level Python web framework for rapid system development.	https://www.djangoproject.com/
Matplotlib	Matplotlib is an object-oriented API (Application Programming Interface) for embedding plots into applications.	https://matplotlib.org/
Scikit-learn	Scikit-learn is open-source data analysis library, and Machine Learning library that contains numerous model algorithm.	https://scikit-learn.org/stable/

### Experimentation of the recommender tool

**Fig. 3:**
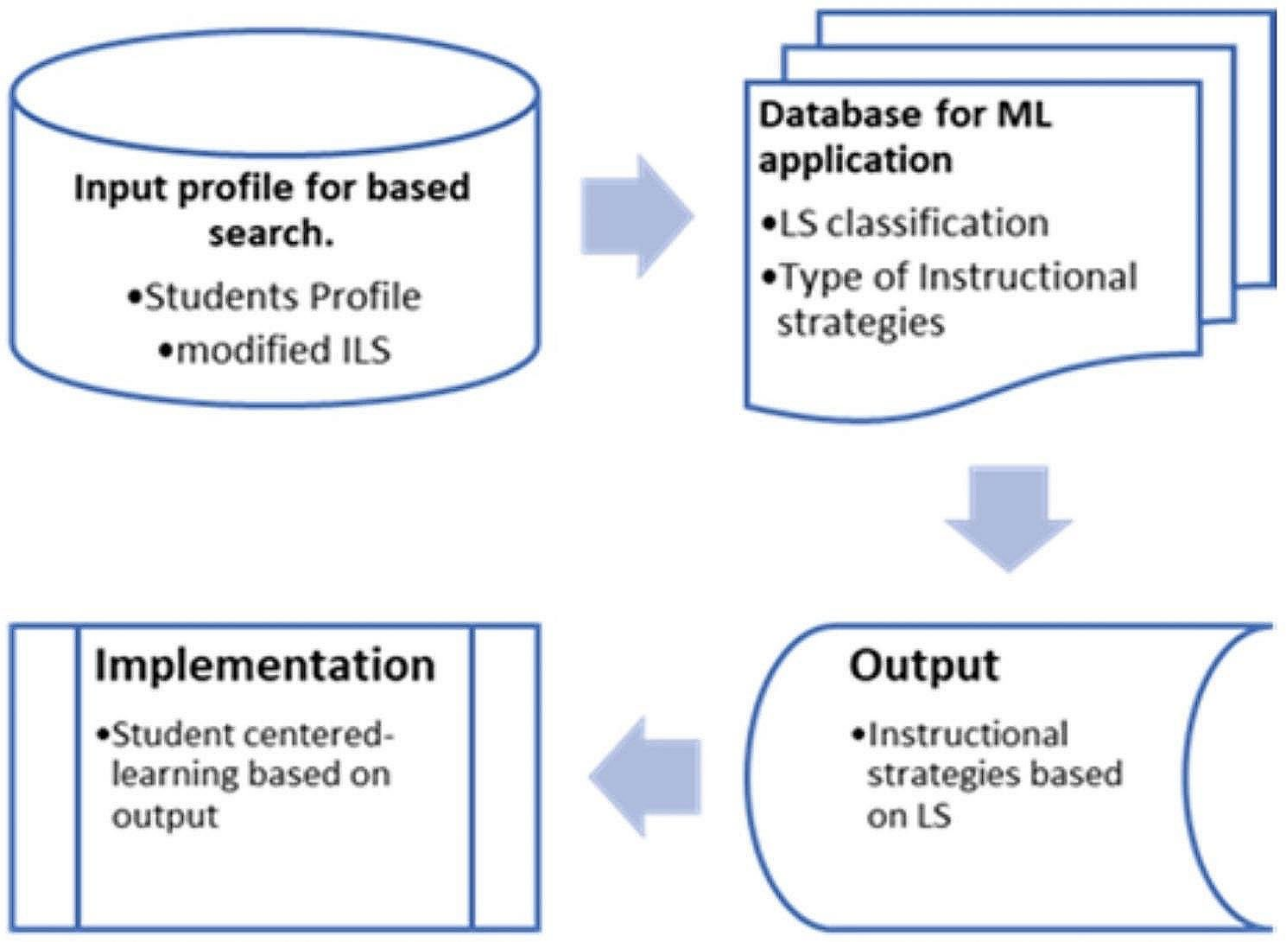
Experimentation of IS recommender tool

The experimentation of the processes was according to the following sequence (Figure: 3):

1. Students must register on the online database of this research.
2. Students answered the web-based m-ILS.
3. The pool of dataset fed to the decision tree model for calculation and extraction of students’ answers, resulting in automatic classification of students’ LS dimensions.
4. Output would be the automated mapping of students’ LS to the suitable IS for the students.

### Accuracy of recommender tool

Confusion matrix was used to evaluate the decisiontree machine learning algorithm’s accuracy for the given dataset. Simultaneously, it evaluated the classification model’s performance. It summarises the model’s predictions and compares them to the true data labels. The evaluation results were based on four different values: true positives (TP)-: the model correctly predicted a positive class, false positives (FP) - the model predicted a positive class, but the true label was negative, true negatives (TN)- the model correctly predicted a negative class, and false negatives (FN)- the model predicted a negative class, but the true label was positive.

These values are then used to calculate various performance metrics of the classification model of scikit-learn in Python, namely accuracy, precision, recall, and F1 score. Below are the examples:

- Accuracy = (TP + TN) / (TP + FP + TN + FN)
- Precision = TP / (TP + FP)
- Recall (or Sensitivity) = TP / (TP + FN)
- F1 Score = 2 * (Precision * Recall) / (Precision + Recall)

Recall (or sensitivity) determines model’s ability to accurately classify students’ LS after answering the m-ILS questionnaire.

  Specificity = TN / TN + FP

Specificity is referred to as true negative rate. As seen in the equation above should be the proportion of true negative (TN) to true negative and false positive (FP). It requires the ability to recognize with accuracy as part of the recommender tool in classifying students’ LS.

## Results

### Decision tree model accuracy and confusion matrix result

The initial dataset of 50 students to train the decision tree ML models showed relatively low accuracy due to human error in the annotation (Table 3). Following the creation of simple rule-based program for automated calculation of the student LS’s score and annotation, an increased number of 255 dataset was used to train and test the recommender system.

**Table 3:** Classification accuracy of different learning styles after feature engineering

Learning Style	Dataset	Model	Accuracy
Processing	**N = 50**	Decision tree	62%
Perception			85%
Input			54%
Understanding			46%
Processing	**N = 255**	Decision tree	100%
Perception			100%
Input			100%
Understanding			100%

**Fig. 4:**
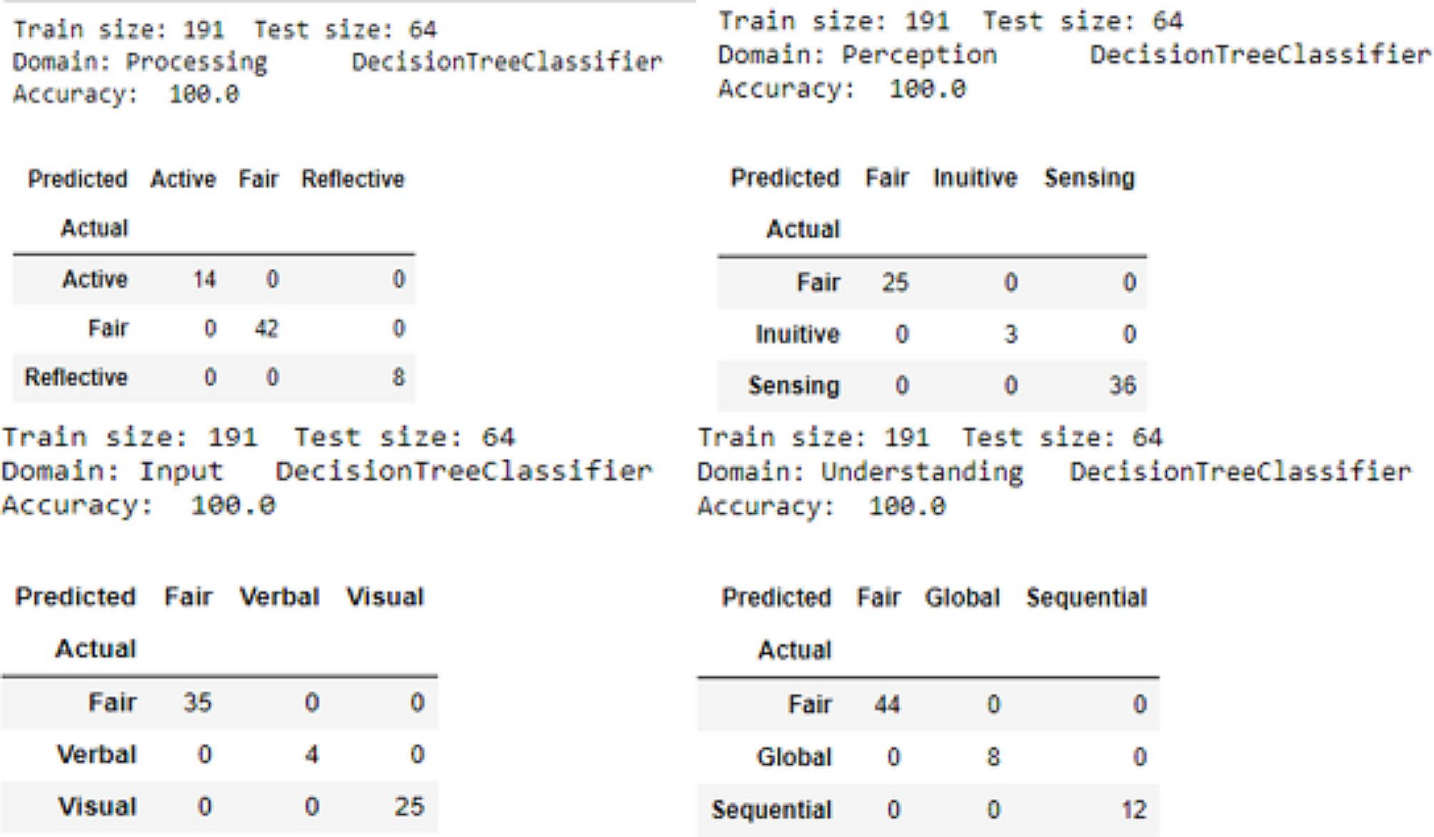
Confusion matrix for decision tree model empowered recommender system

In the multiclass confusion matrix, the diagonal elements represent the number of correct predictions for each type of LS (Fig. 4). Using the decision tree model, a total of 64 samples were correctly predicted for the whole total of the test samples. Therefore, in this study the diagonal elements showed the expected outcome, showing that the model is performing well and accurately predicting the class labels for each LS classification. Thus, the overall accuracy for the recommender tool was 100%.

**Fig. 5:**
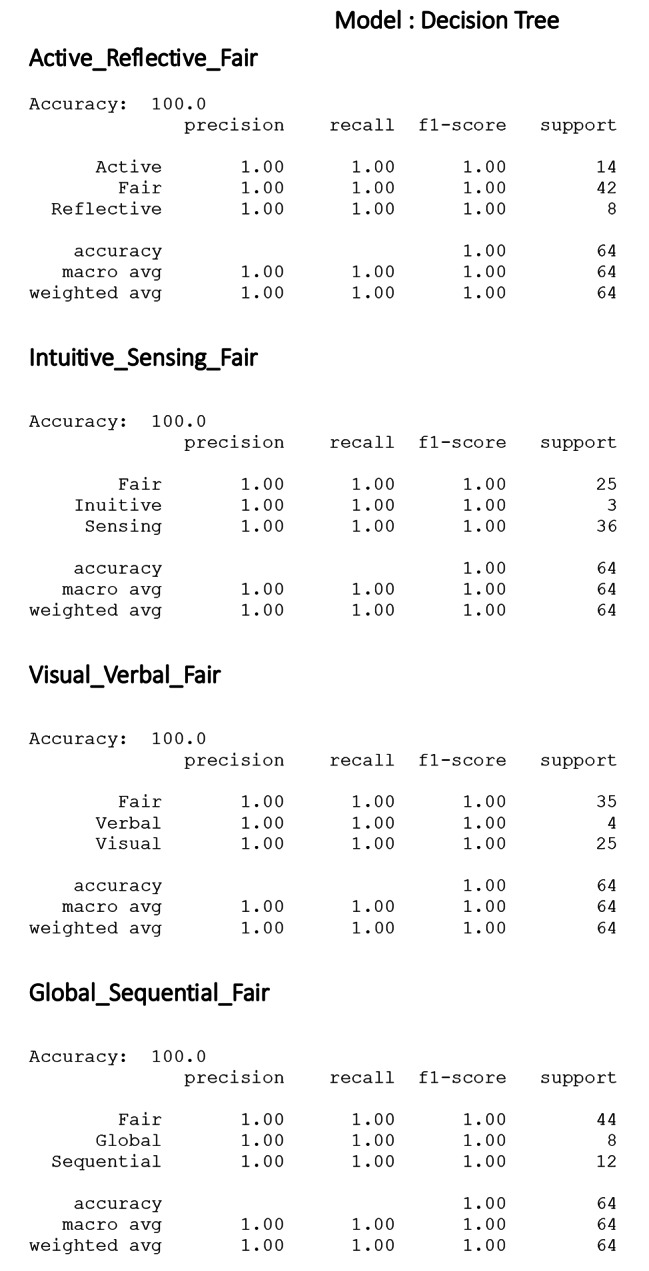
report of classification model performance metrics

The value of accuracy, precision, recall, and F1 score are shown in Fig. 5. For the recommender system using the decision tree model showed a perfect F1 score of 1.0, indicating perfect precision and recall, reflecting a substantial value of sensitivity and specificity.

### The decision tree model visualization

**Fig. 6:**
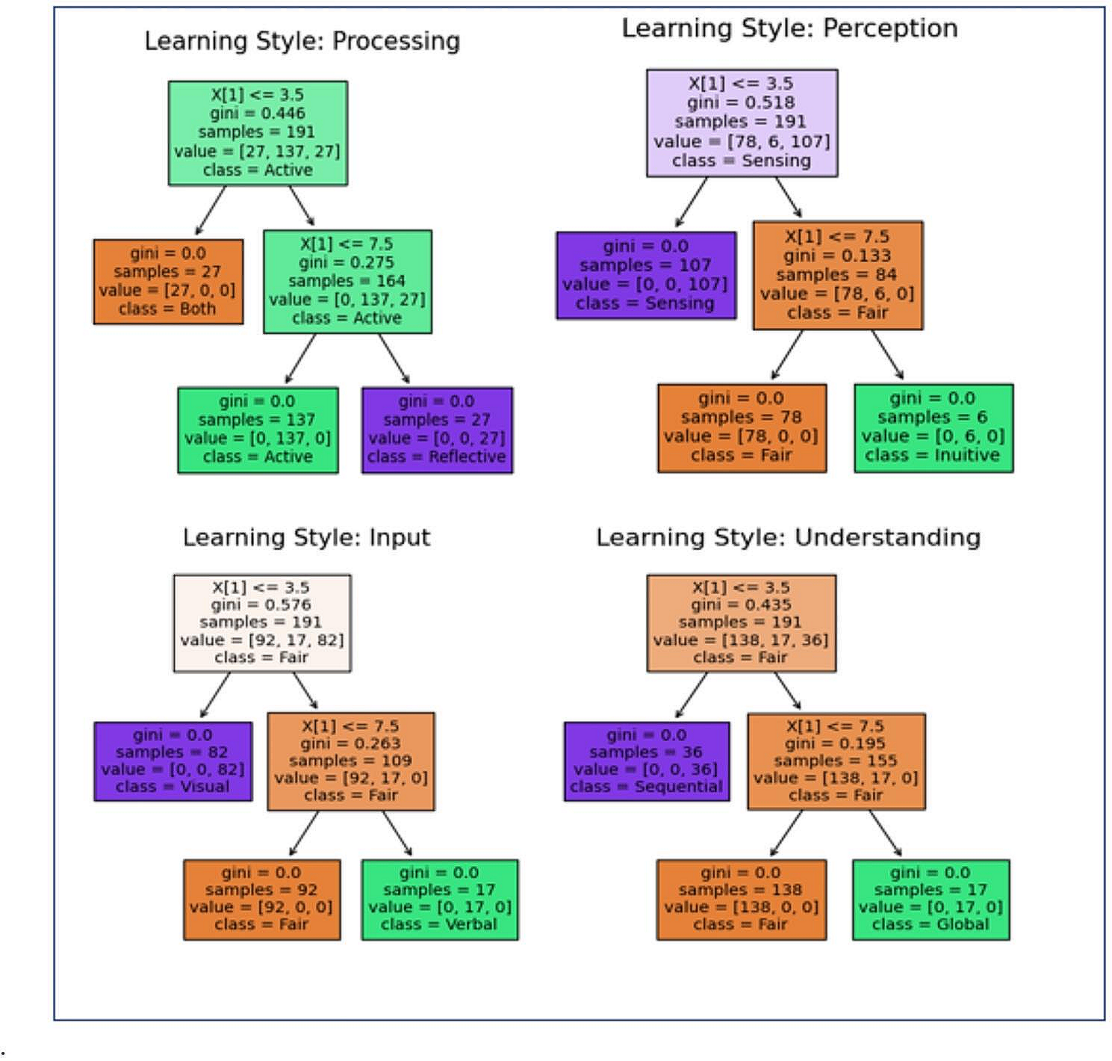
Visualization for Decision Tree model’s rule for Learning Style Dimensions (*N = 255*)

Figure 6 shows the visualization of the decision tree model after the training and testing were completed. Comparing side by side, the decision tree model that was trained with fewer features showed higher accuracy and less complex model visualization. This demonstrated that feature engineering that led to feature reduction was a vital phase to improve the effectiveness of the model.

Through the application of decision tree supervised learning, automation of the mapping between LS (input), and IS (target output) was generated instantly with detailed information of each LS.

**Fig. 7:**
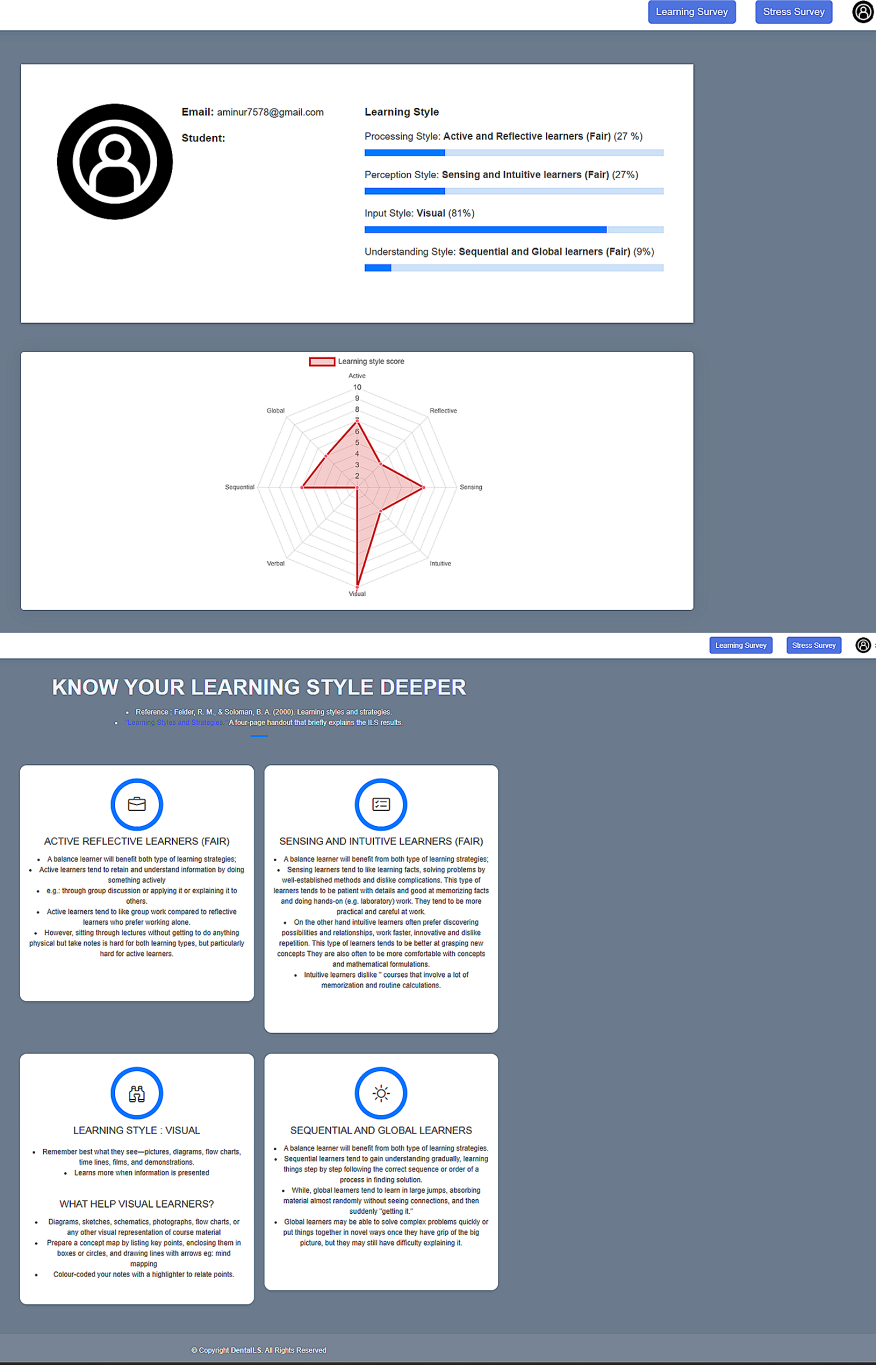
Results with detailed information of each LS for individual students

### Learning styles analysis

Results indicated that 34.9% of the 255 students were prone with one (1) LS preference. The majority of 54.3% were with two or more LS preferences. Students with fairly balanced LS was reported at 12.2% (Table 4). Apart from the eight main LS, there were 34 combinations of LS classifications for the dental students of Universiti Malaya. Among others are Sensing, visual and the combination of sensing visual were the main LS reflected by the students (Fig. 7).

**Table 4:** Distribution of dental undergraduates prone LS preference

Learning Styles		N	Valid Percent	Cumulative Percent
**1LS**	Active	6	2.4	
	Global	2	0.8	
	Intuitive	3	1.2	
	Reflective	12	4.7	
	Sensing	35	13.7	
	Sequential	8	3.1	
	Verbal	1	0.4	
	Visual	22	8.6	34.9(34.9)
2 LS	Active Sensing	4	1.6	
	Active Sequential	1	0.4	
	Active Verbal	1	0.4	
	Active Visual	6	2.4	
	Intuitive Global	1	0.4	
	Intuitive Verbal	1	0.4	
	Reflective Global	1	0.4	
	Reflective Intuitive	1	0.4	
	Reflective sensing	9	3.5	
	Reflective Verbal	1	0.4	
	Reflective Visual	3	1.2	
	Sensing Global	1	0.4	
	Sensing sequential	15	5.9	
	Sensing Verbal	4	1.6	
	Sensing Visual	31	12.2	
	Verbal Sequential	3	1.2	
	Visual Global	3	1.2	
	Visual Sequential	3	1.2	35.2(70.1)
3 LS	Active Intuitive Visual	1	0.4	
	Active Sensing Sequential	3	1.2	
	Active Sensing Verbal	1	0.4	
	Active Sensing Visual	9	3.5	
	Active Visual Global	1	0.4	
	Reflective Sensing Sequential	1	0.4	
	Reflective Sensing Verbal	3	1.2	
	Reflective Sensing Visual	2	0.8	
	Sensing Reflective Visual	1	0.4	
	Sensing Verbal Global	2	0.8	
	Sensing Visual Sequential	6	2.4	14.1(84.2)
4LS	Active Sensing Visual Global	1	0.4	
	Active Sensing Visual Sequential	5	2	
	Reflective Intuitive Verbal Global	1	0.4	
	Reflective Sensing Verbal Sequential	1	0.4	
	Reflective Sensing Visual Sequential	1	0.4	3.6 (87.8)
	Balanced	31	12.2	12.2(100.0)

From Table 4, most students are prone to have either sensing (13.7%) or visual (8.6%) LS. The combination of sensing and visual (sensing visual LS) was reported within 12.2% of the students. These findings indicated that the students prefer to learn and remember following a concrete and detailed procedure through a well-established method and are careful in nature. At the same time, they appreciated learning by seeing (utilising diagrams, charts etc.) and tend to discuss and apply the information either in a group or by themselves.

## Discussion

This study presents an overview of the machine-learning technique used in data mining, focusing on instant and precise prediction of students LS, and recommending suitable IS. The application of decision tree model identified factors most closely related to their LS and learning experience. It is a type of supervised machine learning algorithm that uses a tree-like structure to classify data by splitting the dataset into subcategories based on specific criteria. It works by recursively splitting the input data into subsets based on the values of one of the input features at each internal node, until a decision is made at a leaf node.

The internal nodes of the decision tree represent decisions based on input features from the m-ILS questions, whilst the leaf nodes represent the final prediction of LS classifications. From the entire process of the study, the hierarchical structure of the decision tree interpreting and visualizing the decision-making process was easily understood by looking at the relationship between input features and output prediction.

In computer science and engineering, ML algorithms have been widely used to predict students’ performance based on their admission test scores [21], demographic information and learning behaviors [22]. These studies reported that the algorithm accurately predicted students’ achievement and helped them identify students at risk of struggling academically.

Application of ML algorithm in developing a virtual patient simulator for dental training has been reported. The simulator was able to reproduce the physiological responses of real patients accurately and could be used to train dental students in a safe and controlled environment [23]. A few other studies suggested that ML algorithms may have the potential to improve the quality and efficiency of dental and health education, as well as patient care. ML algorithms have been utilized to assist in making diagnoses of dental diseases based on datasets such as symptoms and characteristics of the patients [24, 25]. Whilst other studies have explored the use of ML algorithms for tasks such as predicting patient outcomes, identifying at-risk patients, developing personalized treatment plans [26], periodontal management [27] and caries management [25].

Although there are published reports on the use of ML in the field of dentistry, its utilisation in dental education is still limited. Therefore, the present study aimed to employ the decision tree model to identify factors most closely related to dental students LS and IS.

Results of the present study showed high accuracy with perfect precision of the developed recommender tool which suggest that educators could potentially be able to benefit from the tool. By leveraging data-driven classification processes, it can provide personalized recommendations, and can improve the educational experience and outcomes for both educators and students. In which, the information obtained from the recommender tool can resolve the conflict between teachers’ preferred teaching methods and students’ learning needs. For instance, through the recommender tool’s automated output, the time taken to identify students’ LS and mapping to suitable IS will significantly reduce. As a result, suitable learning activities and learning materials can be organized. This could help to nurture students’ positive learning behavior as well as their ability to focus. A study reported that providing students with learning materials and teaching activities that match their preferred LS led to greater potential assisting the student to integrate, process and enjoy learning in numerous ways [12]. Studies also reported that apart from improving students’ participation in class, learning about students’ LS played a crucial role in improving teaching practices and communication with students [28, 29].

However, as with any modern technology, there are challenges and limitations. These include issues related to data privacy, bias, and fairness; and the need for specialised skills and resources to develop and implement the ML algorithms in dental education. Nonetheless, the growing interest and research in this area suggest that there is potential for ML technique to make a positive impact in both dental education and dental health services.

Results of the present study demonstrated reflected that half of the dental students presented with tendency of having ‘sensing’ LS. This type of learners prefers facts and concrete examples, practical orientation, patience with details and ‘visual’ LS preferences in which students prefer to use images, graphics, colours, and maps to communicate ideas and thoughts. The present findings agreed with other studies that used ILS to assess the LS of dental and medical students, in which the majority of their students were characteristically prone to have sensing and visual LS [12, 30]. Dalmolin and others suggested that by making the students aware of their LS enable them to unleash their learning potential. Researchers claimed that when educators are well informed of students’ LS, various teaching approaches and activities can be done and this will lead to improvement of students’ performance and learning experience [12, 31, 32]. Other studies indicated that the realignment of students’ LS also showed improvement in students’ learning experience and performance following changes in learning modalities based on students’ own LS [13, 33].

Opinions among educators may differ regarding the implementation of instructional strategies based on students’ LS. While some see the benefits of such an approach, including professional development opportunities, mentorship, and a supportive community, others may have concerns about time and institutional support. Striving for balance is key to shaping attitudes toward student-centred approaches. Higher education authorities, like university administrators, can play an essential role in driving positive changes by embracing innovative practices and supporting faculty development [34]. To create a higher education system that is truly dynamic and responsive, authorities must take bold steps such as implementing policy changes, allocating resources for technology integration, and establishing frameworks that promote student-centred approaches. These measures are essential to achieve the desired outcome. A recent study on differentiated instruction in learning clearly highlights that successful implementation of differentiated instruction requires consistent training and development opportunities for educators [35].

This tool provides valuable support to dental educators who would like to adopt a student-centred approach in the planning of their teaching and learning activities that would suit students’. However, this study is only limited to the use of decision tree ML model. In the future, more data should be collected and comparison of the performance of different machine learning models should be carried out to compare the accuracy, reliability, and precision of the recommender tool. Furthermore, it is also important to consider other factors, such as model complexity and interpretation, when choosing the most suitable ML approach for a given task.

### Limitations

The limitation of this study is it focuses only on the mapping of LS and IS for dental students. Therefore, the developed referral system will only propose suitable for dental students. Changes will be required to use in general higher education students.

## Conclusion

The newly developed recommender tool empowered by ML with the ability to instantly classify students’ LS and map to suitable IS, is the first educational program in dentistry to facilitate dental educators in the planning of suitable teaching and learning activities. By leveraging data-driven classification processes, it can provide personalized recommendations, save time, enhance instructional strategies, support targeted interventions, and facilitate continuous professional development. Its application will help to promote the employment of a student-centered approach in dental education.

## Data Availability

The data is available from the corresponding author, upon reasonable request.

## Abbreviations

FSLSM: Felder-Silverman Learning Style Model
ILS: Index of Learning Styles
IS: Instructional strategies
LS: Learning style
m: ILS-modified index of learning styles
ML: Machine learning
SCL: Student-centred learning
