# Correction: Utilizing decision tree machine learning model to map dental students’ preferred learning styles with suitable instructional strategies

**DOI:** 10.1186/s12909-024-05162-2

**Published:** 2024-02-23

**Authors:** Lily Azura Shoaib, Syarida Hasnur Safii, Norisma Idris, Ruhaya Hussin, Muhamad Amin Hakim Sazali

**Affiliations:** 1 Department of Paediatric Dentistry & Orthodontics, Faculty of Dentistry, Universiti Malaya, 50603 Kuala Lumpur, Malaysia; 2 Department of Restorative Dentistry, Faculty of Dentistry, Universiti Malaya, 50603 Kuala Lumpur, Malaysia; 3 Department of Artificial Intelligence, Faculty of Computer Science and Information Technology, Universiti Malaya, 50603 Kuala Lumpur, Malaysia; 4 Department of Psychology, International Islamic University Malaysia, Jalan Gombak, 53100 Kuala Lumpur, Malaysia; 5 Department of Artificial Intelligence, Faculty of Computer Science and Information Technology, Universiti Malaya, 53100 Kuala Lumpur, Malaysia

**Correction: BMC Medical Education (2024) 24:58**

10.1186/s12909-023-05022-5

Following publication of the original article [1], we have been informed that the title has a spelling.

The incorrect title is: “Utilizing decision tree machine model to map dental students’ preferred learning styles with suitable instructional strategies.”

The correct title is: “Utilizing decision tree machine learning model to map dental students’ preferred learning styles with suitable instructional strategies.”

The original article has been corrected.
